# Long-Acting Atypical Antipsychotics: Characterization of the Local Tissue Response

**DOI:** 10.1007/s11095-014-1308-4

**Published:** 2014-02-21

**Authors:** Sara Montminy Paquette, Had Dawit, Magali B. Hickey, Elaine Merisko-Liversidge, Örn Almarsson, Daniel R. Deaver

**Affiliations:** Alkermes, Inc., 852 Winter Street, Waltham, Massachusetts 02451 USA

**Keywords:** atypical antipsychotics, long acting injectables, foreign body reaction

## Abstract

**Purpose:**

Long-acting injectables (LAIs) are increasingly recognized as an effective therapeutic approach for treating chronic conditions. Many LAIs are formulated to create a poorly soluble depot from which the active agent is delivered over time. This long residing depot can cause localized chronic-active inflammation in the tissue, which has not been well defined in the literature. The purpose of this work is to establish an experimental baseline for describing these responses.

**Methods:**

Non-human primates and rodents were used to examine the response to LAI formulations of two clinically relevant atypical antipsychotics, aripiprazole monohydrate and olanzapine pamoate monohydrate.

**Results:**

A foreign body response develops with elevations of key cytokines such as IL-1α, IL-1β, TNFα, and IL6 at the site of injection. However, the tissue response for the two atypical antipsychotics compounds diverge as evidenced by quantitative differences observed in cytokine levels at various time points after dosing.

**Conclusions:**

Our studies show that, while the drugs are in the same therapeutic class, the response to each of these compounds can be distinguished qualitatively and quantitatively, supporting the idea that the injection site reaction involves a multiplicity of factors including the properties of the compound and cellular dynamics at the site of injection.

## INTRODUCTION

The key to optimal efficacy of many drugs is sustained systemic exposure to therapeutic concentrations of the active compound. Long-acting injectable (LAI) drug products are designed to provide this prolonged exposure. LAIs are usually delivered either subcutaneously or intramuscularly, enabling slow drug release over an extended period of time (1). There are a wide variety of approaches used for LAI delivery including microsphere technology, liposomal encapsulation, nano- and microcrystalline dispersions, lipophilic solutions, oil-based emulsions, and implants. LAIs are available for a wide range of medical indications, and interest in this area continues to grow because of LAIs impact on adherence and improved patient outcome (1). The area of atypical antipsychotics (ATAPs), which are used in the treatment of schizophrenia, bipolar disorder and depression, has seen major advances in recent years including LAIs of important drugs like risperidone, palperidone, olanzapine and aripiprazole (2–5).

While the LAI approach can provide sustained release over weeks or months, the deposition of foreign materials in the tissue bed can result in a variety of tissue responses (6, 7), which often are characterized clinically as an injection site reaction (ISR) or injection site granuloma. ISRs encompass a broad range of reactions from minor events such as pain, induration and redness to more significant tissue responses including nodule or cyst formation and sterile abscesses (2). Granulomas are strictly defined as nodular inflammatory lesions, usually small or granular, firm, persistent, and containing compactly grouped mononuclear phagocytes (8). Pathologically, granulomas are compact, organized collection of mature mononuclear phagocytes, not necessarily accompanied by accessory features such as necrosis(9). However, the term granuloma is often used to describe any nodule formation, and is frequently used in descriptions of ISR’s.

There are several case reports in the literature documenting sterile abscess formation with long-acting gonadotrophin-releasing hormone agonist leuprolide, resulting in resistance to treatment in some cases (10–12). Subcutaneous injection of enfuvirtide, an antiviral treatment for HIV, results in granuloma formation at the site of injection in a large proportion of patients, as determined by pathological analysis (13, 14). Adalimumab, used in the treatment of rheumatoid arthritis and other autoimmune disorders, is reported to cause lesions and interstitial granulomatous drug reactions (15). Despite these case reports, detailed reports describing the ISR and local tissue response to LAIs are scarce, with minimal characterization of the local cellular and cytokine changes to these responses.

Atypical antipsychotics, also known as second-generation antipsychotics, have an array of pharmacologic actions including blockade of dopamine receptors. They also tend to have high efficacy in patients without causing a number of the side effects seen with first-generation antipsychotics, such as extrapyramidal motor control disabilities (16). LAI formulations of ATAPs have been recently developed for the treatment of these disorders. These include risperidone (Risperdal® Consta®), a polymer microsphere formulation first approved in 2002, a nano-crystalline formulation of paliperidone palmitate (Invega® Sustenna®), approved in 2009, olanzapine pamoate monohydrate (Zyprexa Relprevv®) also approved in 2009, and most recently, aripiprazole monohydrate (Abilify® Maintena™) was approved in 2013. These products provide continual exposure to therapeutic concentrations in systemic circulation over weeks and months with reduced peaks and troughs in plasma drug concentration. Additionally, LAIs for these medications are administered by health care professionals to ensure compliance and improve therapy for schizophrenia, bipolar disorder and depression (1, 17–20).

As with the other LAIs, these formulations of antipsychotics may also result in clinically defined ISRs. Depot oil-based injections of the first-generation antipsychotic haloperidol (Haldol® Decanoate®) cause injection site reactions, leading some patients to discontinue treatment (21, 22). LAI formulations of atypical antipsychotics risperidone and paliperidone palmitate have reduced incidences of ISRs compared to haloperidol decanoate, possibly due the fact that these are aqueous-based injections. Nevertheless, redness, pain, swelling, and nodule formation at the site of injection have been reported with both these compounds (3, 23–26). The LAI formulation of olanzapine pamoate monohydrate (Zyprexa Relprevv®) has an incidence of injection site reactions of 8.4%, similar to what was reported with haloperidol (4, 27, 28)*.* Aripiprazole monohydrate (Abilify® Maintena™), recently approved by the U.S. Food and Drug Administration (FDA)-as an extended release formulation, has a reported injection site-related adverse events of 6.3%, with investigator evaluation noting injections site pain, redness, swelling, and induration in 4–26% of subjects in open label stabilization phase studies *(38)*, (29). The work described in this paper was undertaken to characterize the immunological and cellular changes associated with the administration of aripiprazole monohydrate and olanzapine pamoate monohydrate in both non human primates and rodents. The findings are characterized by the histopathology of the FBR resulting from the depot injections in both models and by monitoring the release of various cytokines after dosing in rodents.

## MATERIALS AND METHODS

### Preparation and Formulation of Compounds

Aripiprazole monohydrate was prepared by adding aripiprazole anhydrous free base (Matrix Labs, India) to a 0.2% polysorbate 20 solution (JT Baker, Philipsburg NJ) in phosphate buffered saline (Sigma, St Louis MO). The resulting mixture was left stirring for several days to ensure complete conversion to aripiprazole monohydrate. The solids were then collected using a Hirsch funnel, washed with water and dried *in vacuo* to give aripiprazole monohydrate as a colorless solid. Particle size distribution was measured using a Horiba LA910 to give average volume metrics of d_v_10 = 1 μm, d_v_50 = 5 μm, d_v_90 = 13 μm. Full characterization, including powder X-ray diffraction, fourier transform infrared spectroscopy, differential scanning calorimetry, and thermogravimetric analysis was also conducted.

Olanzapine pamoate monohydrate was prepared by adding olanzapine-free base (Neuland Lab Limited, India) to dimethyl sulfoxide (DMSO) (Sigma, St Louis MO) in a flask. The resulting mixture was shaken and sonicated to ensure dissolution of the solids. The solution was then added to a separate flask containing pamoic acid (Sigma, St Louis MO). The flask containing olanzapine was then washed with twice with DMSO and all contents were combined. The organic solution containing olanzapine and pamoic acid was then added to another flask containing water. The resulting mixture was heated to 40°C and stirred for 20 min. The solution was then cooled to 25°C and the resulting precipitate was collected using a Hirsch funnel and washed with water. The powder was dried *in vacuo* at 50°C overnight to yield olanzapine pamoate monohydrate as a yellow solid. Particle size distribution was measured to give average volume metrics of d_v_10 = 2.8 μm, d_v_50 = 7.4 μm, d_v_90 = 16.0 μm, and full characterization, including powder X-ray diffraction, fourier transform infrared spectroscopy, differential scanning calorimetry, and thermogravimetric analysis was also conducted.

The formulations of aripiprazole monohydrate and olanzapine pamoate monohydrate used in animal studies were prepared to provide 100 and 300 mg/ml suspension by adding either 3 g or 9 g to a 50 ml glass vial. To the solids was added 30 ml of injection vehicle (2–3% sodium CMC, 0.2% polysorbate 20 in phosphate buffered saline (PBS) pH 7). The resulting mixture was sonicated for 10 min and left standing. The contents of the vial were then shaken until a uniform; clump-free suspension was obtained prior to dosing.

### Animal Studies

#### Rodents

Rodents were housed and maintained following the recommendations of the Institute of Laboratory Animal Resources Guide for the Care and Use of Laboratory Animals, 7th Ed. (30) . All experiments were approved by Alkermes’ IACUC. Sprague Dawley rats were obtained from Charles River Laboratory between 6 to 8 weeks of age and weighed between 225 and 250 g. Animals were housed two per cage with environmental enrichment, at 64–76 F and using a 12-h light/12-h dark cycle. Food and water were provided *ad libitum*. Rats were allowed to acclimate to housing for at least 1 week after arrival in the facility before being assigned to a study.

#### Cynomolgus Monkey

Single dose intramuscular or subcutaneous toxicology study in cynomolgus monkeys (*Macaca facicularis*) was conducted by an external research organization in accordance with the US FDA Good Laboratory Practice Regulations (Part 58 of 21 CFR), following the recommendations of the Institute of Laboratory Animal Resources Guide for the Care and Use of Laboratory Animals, National Academy Press 1996, and in accordance with all appropriate parts of the Animal Welfare Act regulations. Animals were pair housed during the study. Primate diet was provided twice daily and suplemented three times a day with fruits and vegetables. Water was provided *ad libitum*.

### Animal Studies

#### Cynomolgus Monkey Study

For this study, 18 male cynomolgus monkeys between the ages of 2.75 and 4 years with a mean weight of 3.2 kg were used. The animals were dosed once either intramuscularly in the gluteal or gastrocnemius muscle group or subcutaneously on the dorsal surface with aripiprazole monohydrate in 3% carboxymethylcellulose, 0.1% polysorbate 20 and 0.9% sodium chloride in sterile water as vehicle (Table I). An additional 4 animals were injected intramuscularly with vehicle only as controls. A volume of 0.5 ml/kg was used for all materials. The animals were then observed for 30 day, after which 16 of the animals were humanely euthanized and necropsied. Animals were sedated with ketamine/xylazine and then euthanized by exsanguination under sodium pentobarbital anesthesia. The remaining 6 animals were further observed for a 20 day period to determine if there was recovery from any effects seen. After this recovery period, the animals were euthanized as described above. A full postmortem examination was conducted on the animals including macroscopic and microscopic pathology.

**Table I:** Experimental Design for Cynomolgus Monkeys

Number of animals	Aripiprazole dose (mg/kg)	Route	30 day	50 day
4	0	IM	4	0
6	15	IM	4	2
6	120	IM	4	2
6	120	SC	4	2

#### Rodent Studies

For experiments conducted to determine threshold dose for localized tissue responses, five groups (*n* = 6 per group) of Sprague Dawley rats were sedated with isoflurane and then injected in the intrascapular region with aripiprazole monohydrate or olanzapine pamoate monohydrate at a dose of 3, 10, 30, 100 or 300 mg/site for each drug using a 21-gauge, 1-in. needle attached to a 1-cc syringe. The dose volume was 1.0 ml for all groups. The animals were continuously monitored until they recovered fully from the anesthesia. Ten days following injection, animals were euthanized by carbon dioxide asphyxiation, and the injection site was excised and weighed. The injection sites were defined as the drug depot and surrounding tissue.

For time course experiments, rats were dosed *via* syringe subcutaneously in the intrascapular region using a 21-gauge needle as described above. On days 1, 3, 7, 10, 14, and 21, three animals per group were euthanized by carbon dioxide asphyxiation. Whole blood was obtained from the animals post mortem by cardiac puncture and up to 10 ml of blood collected. The injection sites were excised and placed on ice for further processing.

### Peripheral Blood Mononuclear Cells and Fluid Isolation

Whole blood from rats was placed in an EDTA Vacutainer® tube (BD, Franklin Lakes NJ, catalog # 366643), mixed thoroughly, and rested at room temperature for 15 min. Blood was then mixed with 1.25 ml of Optiprep™ media (Axis, Oslo Norway catalog #1114542) in a 50 ml conical. One milliliter of Tris-buffered saline (TBS) was layered on top of the blood/Optiprep™ mixture, and spun at 1,200 g for 30 min at room temperature. After centrifugation, the buffy coat was isolated and transferred to a 15 ml conical. The cells were then washed in 10 ml of PBS (Lonza, Walkersville MD), centrifuged again to remove any residual fluid, and were then subjected to red blood cell lysis (RBC lysis buffer, R7757 Sigma-Aldrich, Saint Louis MO). The cells were then washed twice and resuspended at a concentration of 10^7^ cells per milliliter.

Serum was isolated by adding 1 ml of whole blood to Microtainer® serum separator tubes (BD, Franklins Lakes NJ 365956). The blood was incubated on ice for one hour, and then centrifuged at 14,000 × *g* for 3 min. The serum was removed from the tube and immediately frozen at −80°C for later cytokine analysis.

### Processing of Injection Sites and Surrounding Tissue Response

Injection sites were isolated from euthanized animals and immediately placed on ice. Fluid contained within the structure was aspirated with a 21-gauge needle using a 1-cc tuberculin syringe. Immediately following collection, fluid was placed on ice and then frozen at −80°C for cytokine analysis. The injection sites were dissected on ice and then centrifuged at 3,000 × *g* to obtain additional fluid, which was pooled with the original aspirate; typically this yielded no more than 10% of the total recovered volume. The remaining tissue was frozen and stored at −80°C.

### Cytokine Analysis

Cytokines levels were measured in both sera and fluid obtained from the injections site by multiplex analysis using a Bio-Plex Pro™ rat cytokine group 1 custom 13 plex assay which contained the following targets: IL-1α, IL-1β, IL-2, IL-6, IL-10, IL-12p70, IFNγ, GRO/KC, MIP-1α, MIP-3α, RANTES, TNF, and VEGF (Bio-Rad, Hercules CA, Catalog #L8000LED47). The assay protocol provided with the kit was followed for these analyses. Assay plates were processed on a Luminex® 200. Data were analyzed using the xPONANT 3.1 software.

### Determination of Cell Populations

PBMCs from whole blood were isolated as described above. Cells were then resuspended in 100 μL FACS buffer comprised of PBS (Lonza, Walkersvile MD) containing 1% fetal calf serum (FCS) (GIBCO®, Carlsbad, CA catalog #16000-077) and 1 μg of anti-rat CD23 (BD, San Jose CA, catalog #550270) FC block at a concentration of 10^6^ cells/ml. Cells were incubated at 4°C for 15 min and then stained for surface markers to determine cell populations in whole blood. All antibodies were obtained from BD Biosciences (San Jose, CA). Markers examined were RP1 (550002), CD4 (561578), CD8a (558824), HIS48 (554907), HIS 36 (554901), CD11b/c (554861), and CD161 (555009). After staining, the cells were analyzed on an Attune® flow cytometer (Applied Biosystems® Carlsbad, CA).

### Statistical Analysis

Data are expressed as either means ± standard deviation or as individual points with bars indicating the means. Statistical analyses were performed using GraphPad Prism® 5 and SAS 9.2. Two-way analysis of variance (ANOVA) were conducted on cytokine and cell data, with statistical significance established at *p* < 0.05.

## RESULTS

### Intramuscular or Subcutaneous Injections of Aripiprazole Monohydrate Results in Chronic-Active Inflammation in non-Human Primates

It has been reported that depot injections of atypical antipsychotics may cause pain or swelling to occur in patients during clinical trials of many different compounds. To investigate this occurrence, cynomolgus monkeys were injected with aripiprazole monohydrate (APZ) once either subcutaneously with 120 mg/kg, or intramuscularly with 15 mg/kg or 120 mg/kg respectively, and then observed for 30 to 50 days (Table I). As controls, all animals were injected with the vehicle in the contra-lateral leg. Also, a separate control group were injected with vehicle alone and observed for the same period of time. After 30 days, the control animals and 4 animals per test group were humanely euthanized and necropsied, while 2 animals per test group recovered for 20 days before being euthanized (Table I). The injection sites were also subjected to analysis by a pathologist to assess inflammation state. Six days after injection, discrete masses were detected at the injection site in all the animals that had received subcutaneous injections, and were present throughout the test and recovery period. These masses ranged in area from 1.5 cm^2^ to 14 cm^2^. Upon necropsy at day 30 and 50, visible masses were present at the injection site by macroscopic analysis, along with chronic-active inflammation at the injection site as determined by microscopic analysis (Table II). Chronic-active inflammation is defined as infiltrates of foamy macrophages lined by lymphocytic aggregates and central areas of necrosis as determined by a pathologist. In the animals injected subcutaneously, the injection sites also had clear central areas. Pathological analysis determined that this chronic-active inflammation in the subcutaneous group to be marked to severe severity at both time points, indicating that there was an ongoing local immune response to the LAI (Table II).

**Table II:** Incidence of Chronic-Active Inflammation in Cynomolgus Monkeys Following Injection of Aripiprazole Monohydrate

	30 day observations				50 day observations		
Dose (mg/kg)	0	15	120	120	15	120	120
Number of animals examined	4	4	4	4	2	2	2
Route of injection	IM	IM	IM	SC	IM	IM	SC
Minimal	0	0	0	0	0	0	0
Slight	0	1	0	0	1	0	0
Moderate	0	0	1	0	0	0	0
Marked	0	0	1	1	0	0	0
severe	0	0	0	3	0	0	2
Total incidence	**0**	**1**	**2**	**4**	**1**	**0**	**2**

There was also indication of long duration chronic-active inflammation in the animal groups that received intramuscular injection. One animal who received a 120 mg/kg injection had a distinct mass present at the injection site accompanied by hemorrhage, erythema, and ulceration not believed to be due to microbial presence at day 30. This animal was necropsied at day 30, and was seen to have marked chronic inflammation at the site of injection (Table II). A second animal in this dose group had indications of moderate chronic inflammation at the injection site at 30 days, while one in the 15 mg/kg group had slight inflammation at the site (Table II). The data from this study reveled that there was a significant localized reaction occurring at the site of injection with aripiprazole monohydrate, and that it was more pronounced in the subcutaneous space.

### Local Tissue Response Following Subcutaneous Administration of Aripiprazole Monohydrate in Rodents

**Fig. 1:**
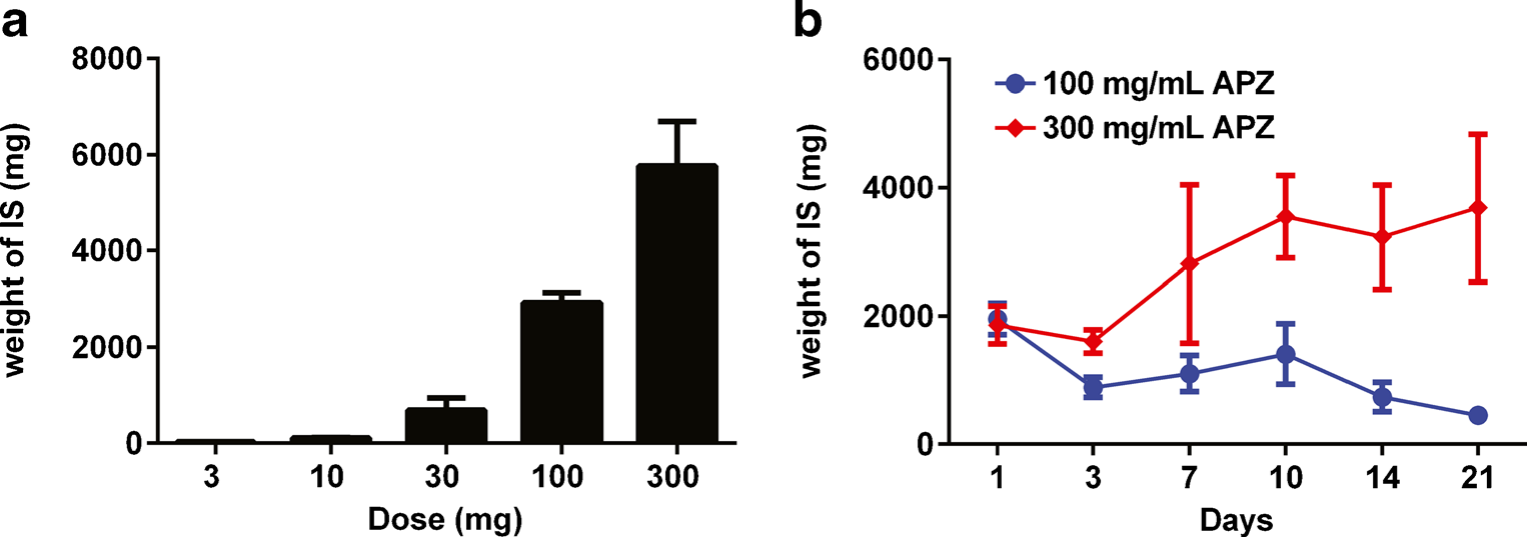
Dose and time response to aripiprazole monohydrate injected subcutaneously in rats. (**a**) Weight of injection site (IS) 10 days following subcutaneous injection of APZ in 3% CMC, 0.1% polysorbate 20, 0.9% NaCl in increasing doses. Average weight is shown in graph with standard deviation (SD), *n* = 6 per dose. (**b**) Weight of the IS over time for two doses of APZ in 2% CMC and 0.2% polysorbate 20. Two-way analysis of variance shows a significant dose (*p* = <0.0001) and dose × time (*p* = 0.0011) effect.

The studies in non-human primates illustrated that there was a significant local tissue response to aripiprazole monohydrate depots, especially in the subcutaneous space. Therefore, further studies were conducted in the subcutaneous space in rodents. In order to determine the dose response curve of local tissue reaction to subcutaneous aripiprazole monohydrate (APZ), increasing doses were administered to rats and the injection sites evaluated 10 days later. The tissue response was characterized by a non-linear dose response curve (Fig. 1a). At doses up to 30 mg, the mass of the injection site, which is defined as the depot and any resultant encapsulating structure, and the surrounding inflamed tissue, was relatively low with a clear inflection point in the response curve occurring at the 100 mg dose. Consequently, additional studies were conducted using 100 and 300 mg of APZ since these doses initiated robust responses.

The response resulting from SC injection of 100 and 300 mg was further characterized over a 21-day period. The weights of the injection sites are shown in Fig. 1b. One day following the injection of APZ, a loose, gelatinous-like material was present surrounding the boundaries of the injection site. By day 3, a well defined fibrous capsule surrounded the site. The weight is significantly affected by the dose (*p* < 0.0001) after 10 days (Fig. 1a). The dose-by-time interaction (*p* = 0.011) is also significant from days 3 to 21, as illustrated in the time courses in Fig. 1b. At the 100 mg dose of APZ, the weights were relatively constant between days 3 and 10 and then began to decrease. In contrast, at the 300 mg dose the weight of the IS increased between days 3 and 10 and then remained constant; the sites did not appear to diminish in size through day 21 (Fig. 1b). A control group that only received injections of vehicle showed no indication of inflammation or encapsulation at any time point, nor was there any fluid present at the site.

**Fig. 2:**
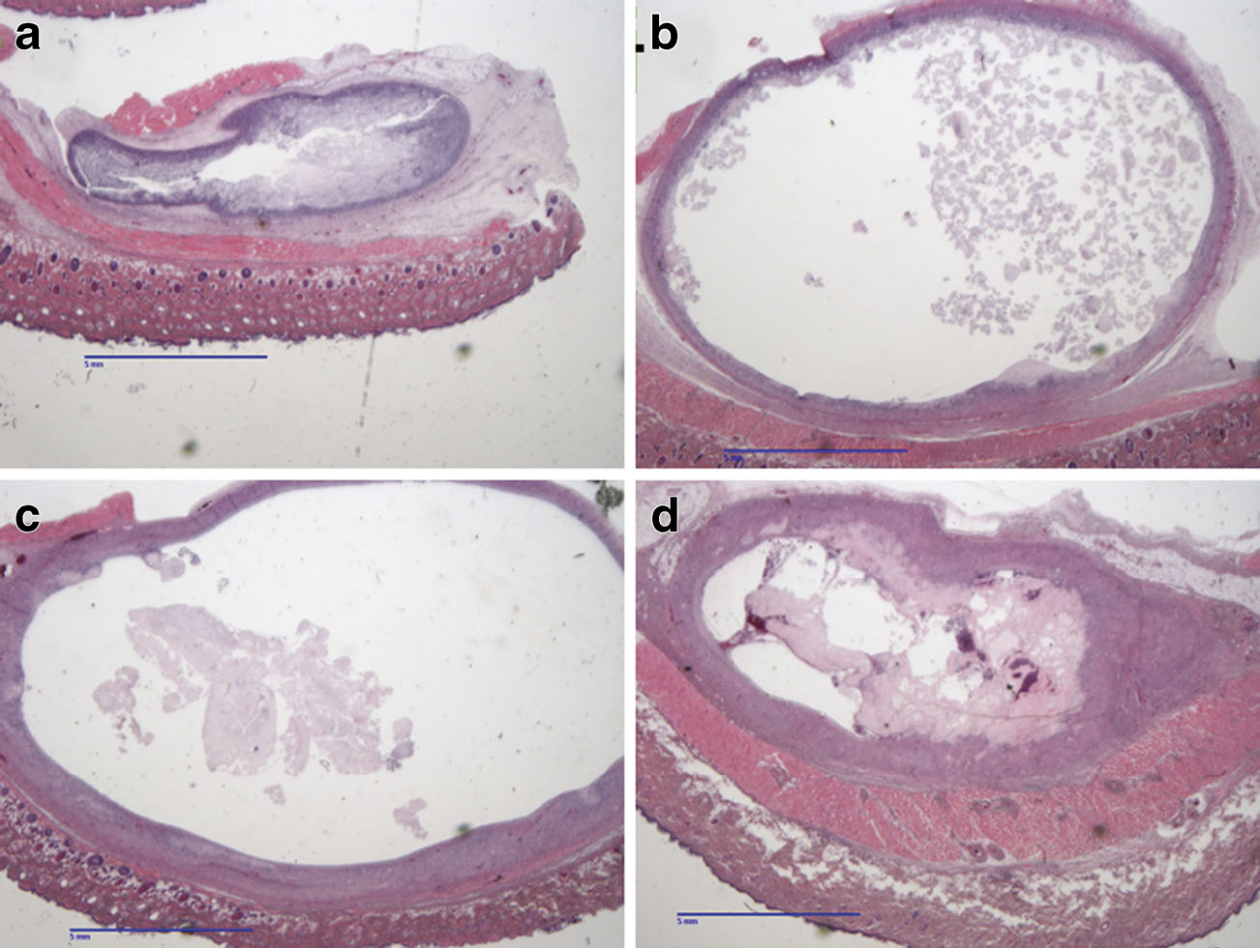
Histological observations of aripiprazole monohydrate injection sites. Micrographs of the IS excised from euthanized rats at (**a**) day 1, (**b**) day 7, (**c**) day 14, and (**d**) day 21 following SC administration of 300 mg of aripiprazole monohydrate. The structures and surrounding tissue are shown at 0.5× magnification following H&E staining. Scale bars are 5 mm. *Slides* are representative of four animals per time point.

While the injection site was less well defined on day 1 (Fig. 2a), it was readily recognizable. By day 7, a distinct fibrous capsule is present surrounding a fluid-filled cavity (Fig. 2b). This encapsulated structure’s wall thickens in this time frame, and extrudate begins to accumulate inside the structure along with cellular debris. This extrudate generally appears yellow and translucent, and contains cellular debris that is either particulate or creamy in consistency. The increase in the mass of the IS between days 7 and 14 is due primarily to the increase in fluid contained inside the structure. There was also significant infiltration of mononuclear cells around the site, indicating a productive immune response (Fig. 2). By day 14, the structures associated with 100 mg of APZ had begun to decrease in size, indicating a process of resolution, with portions of the structure compacting and forming into a nodule and a reduction in the amount of fluid and debris contained within the structure, however with 300 mg doses, they are still increasing in size (Fig. 2c). By day 21, the capsule resulting from SC administration of a 100 mg dose of APZ contained negligible fluid, resulting in a structure containing a firm nodule. When a 300 mg dose is administered, the tissue response surrounding the injection site continues to increase in size up to at least day 21 (Fig. 2d). This increase in size is reflective of the fact that the capsule continues to accumulate fluid inside the structure. The structure wall thins out as the IS increased in size, but nevertheless the wall remains intact and fibrous (Fig. 2d). At day 21, red coloration to the extrudate was also seen inside the structure (Fig. 2d).

### Key Inflammatory Cytokines are Involved in the Foreign Body Response to Aripiprazole

**Fig. 3:**
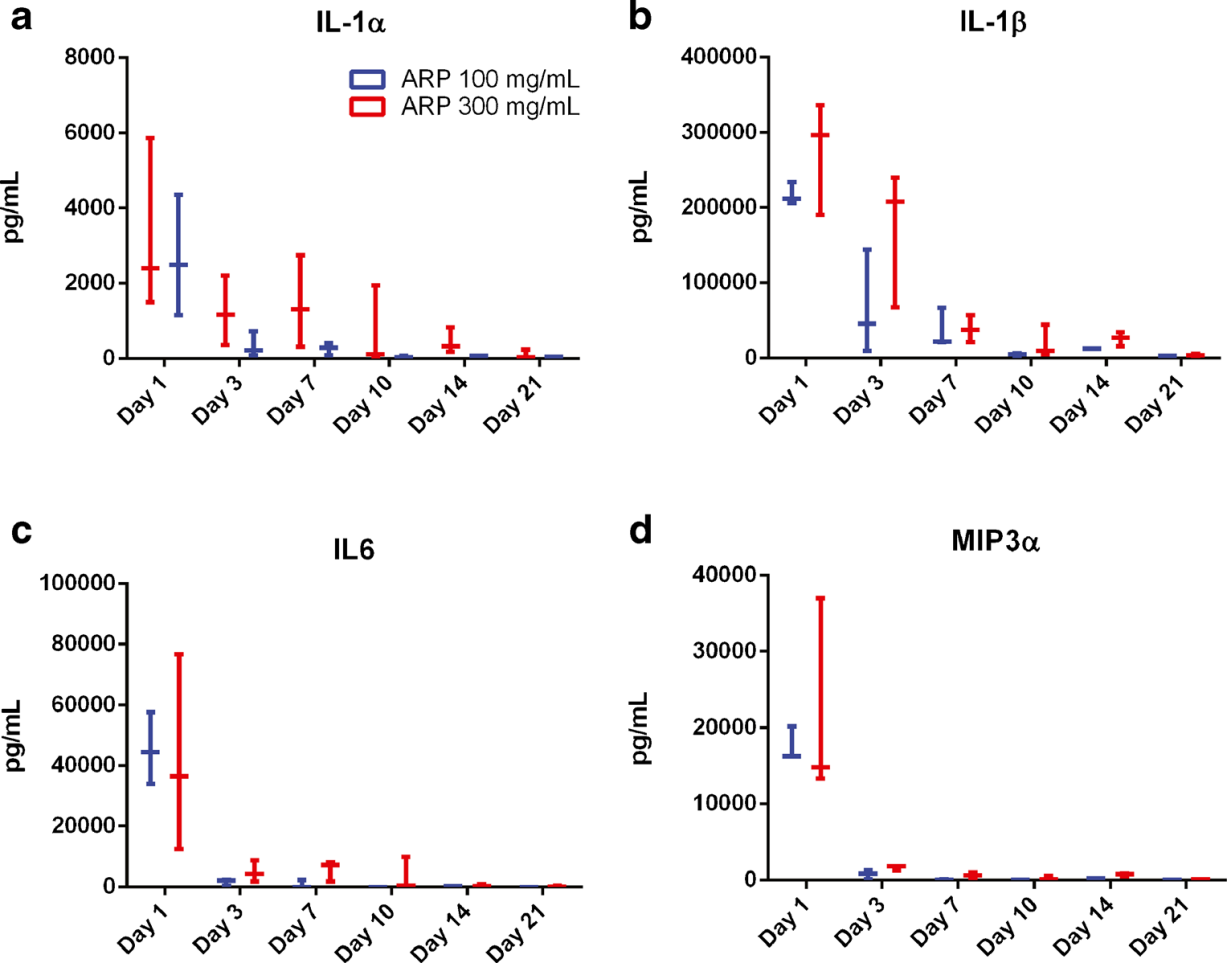
Early cytokines involved in the ISR to aripiprazole monohydrate. (**a**) IL-1α, and (**b**) IL-1β cytokine levels measured in fluid extracted from the encapsulated structure at either 100 mg/ml or 300 mg/ml doses of APZ. Fluid for the analysis was separated from tissue by centrifugation. Cytokine levels from each animal are plotted with bars indicating mean (*n* = 3 per time point and dose). (**c**) IL6 and (**d**) MIP3α cytokine levels were examined in fluid extracted from the encapsulated structure at either 100 mg/ml or 300 mg/ml doses of APZ. Fluid for the analysis was separated from tissue by centrifugation. Cytokine levels from each animal are plotted with bars indicating mean (*n* = 3 per time point and dose).

A significant tissue response following SC injection of APZ has been described. The response displays the key characteristics of a foreign body response (FBR). In order to evaluate the immunological characteristics of this FBR, key inflammatory proteins which may contribute to the formation and maintenance of the tissue response were examined. IL-1α (Fig. 3a) and IL-1β (Fig. 3b), both potent pro-inflammatory cytokines that are key regulators of the early immune response to foreign particles, are present in elevated concentrations by day 1 and then decrease until day 7, after which time they can no longer be detected. IL-6, an important acute-response cytokine secreted by macrophages after injury, and MIP3α, an early chemo-attractant, are detected in the tissue on day 1 only. Neither IL-6 nor MIP3α can be found in significant concentrations on subsequent days (Fig. 3c–d).

**Fig. 4:**
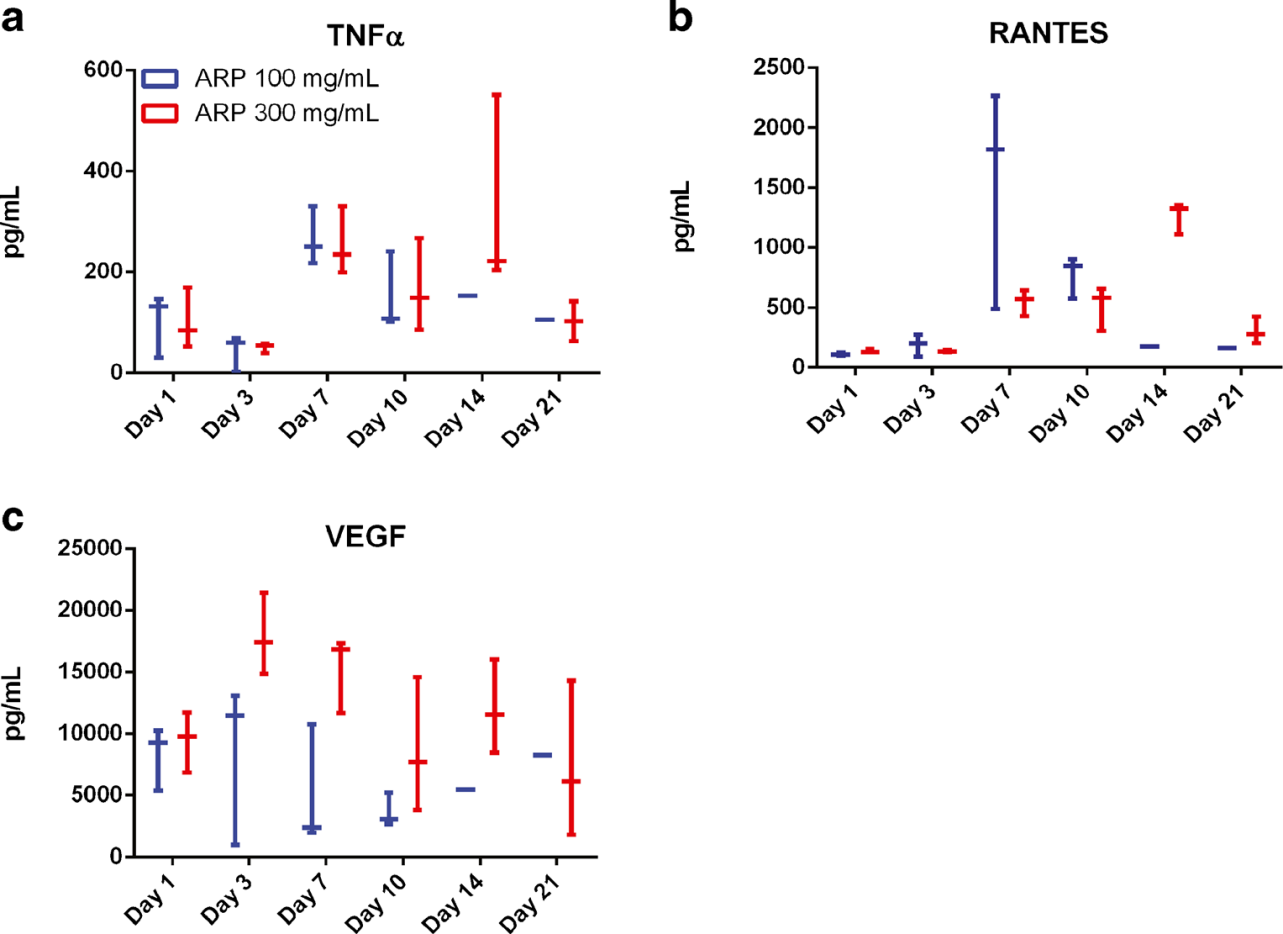
Later cytokines involved in the ISR to aripiprazole monohydrate. (**a**) TNFα is elevated at day 1, then again between days 7 and 14 after SC injection of APZ. Fluid for the analysis was separated from tissue. (**b**) RANTES levels begin to be elevated at day 7, peak between days 10–14, and are reduced by day 21. (**c**) VEGF is elevated in the tissue surrounding the encapsulated structure later in the response time frame. Cytokine levels from each animal are plotted with bars indicating mean (*n* = 3 per time point and dose).

As seen in the time courses of weights of the IS illustrated in Fig. 1b, the response at the depot site continues until at least day 21. During this time, as the immune system continues to respond to the IS, there are changes in the cytokines being expressed at the site, indicating a temporal shift in the type of response being mounted. On day 7, there is elevation of TNFα, which signals through NF-κB and MAPK pathways and has pro- and anti-inflammatory roles (Fig. 4a). RANTES, a chemotactic cytokine important in recruiting lymphocytes and other immune cells to the site of inflammation is also observed (Fig. 4b). Both TNFα and RANTES are integral to the maintenance of the immune response. VEGF, a signal protein that stimulates angiogenesis and vascularization, is additionally elevated (Fig. 4c). VEGF aids in vascularization of the fibrous capsule, and significant levels of this cytokine are observed as early as day 1 and are generally peaking by day 3, though VEGF levels remain high out to 21 days. The presence of these cytokines indicates that there is a significant and sustained FBR to aripiprazole deposited in the subcutaneous space. Indeed, the cytokine profiles helps explain the macroscopic character of the injection sites as they evolve in response to the APZ depot.

### Foreign Body Response Observed with Olanzapine Pamoate Monohydrate Following Subcutaneous Dosing

**Fig. 5:**
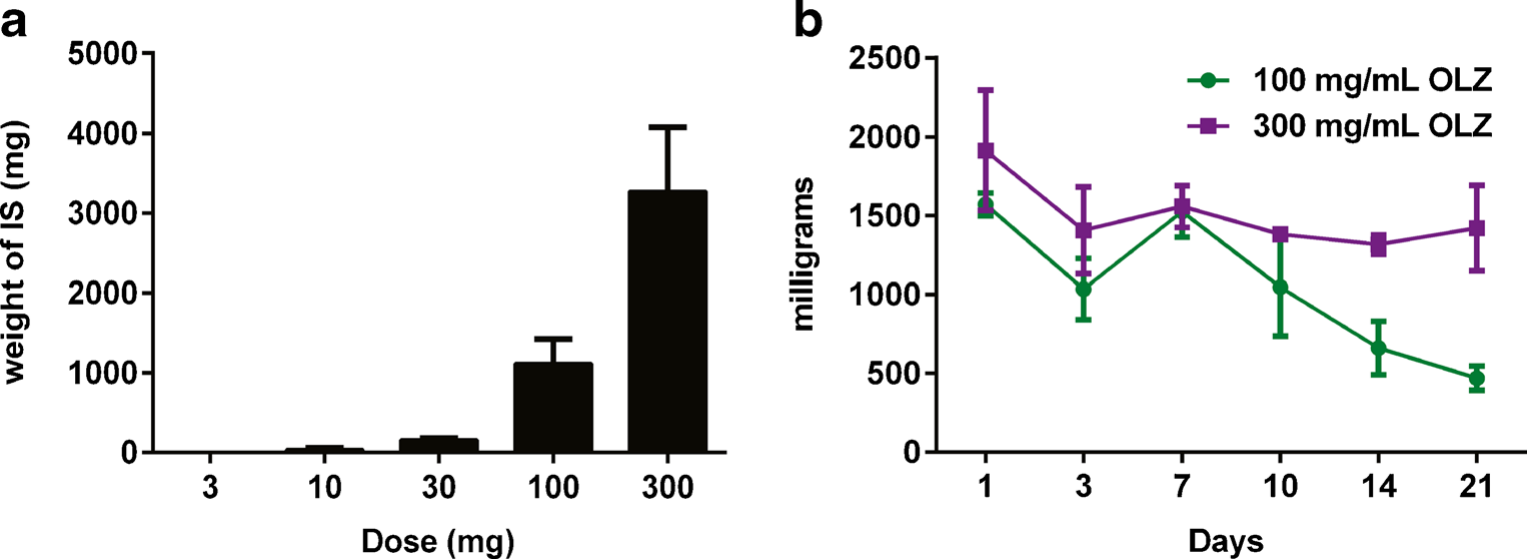
Dose and time response to olanzapine pamoate monohydrate injected subcutaneously in rats. (**a**) Weight of injection site, which includes the encapsulated structure and surrounding tissue, after subcutaneous injections with OLZ in 3% CMC, 0.1% polysorbate 20, 0.9% NaCl at a range of doses at day 10. Average weight of tissue response is shown in graph with standard deviation, *n* = 6 per group. (**b**) Structure weight in response to 100 mg or 300 mg OLZ in 1 ml 2% CMC, 0.2% polysorbate 20 over time. Two way analysis of variance shows a significant dose (*p* = <0.0001), time (*p* = <0.0001), and interaction (*p* = 0.03) effect. The average FBR weight is plotted with *error bars* indicating standard deviation (*n* = 3 per time point and dose).

In order to determine if the tissue response seen with subcutaneous injections of APZ were unique to the compound, analogous studies were conducted with another atypical antipsychotic, olanzapine pamoate monohydrate (OLZ). OLZ was injected subcutaneously into rats as a function of increasing dose. Similar to the situation with APZ SC doses, the threshold dose of OLZ for inducing a significant tissue response was at 100 mg, at which point the weight of the encapsulated structure and surrounding tissue increases significantly compared to what is seen at lower doses (*i.e.* up to 30 mg; Fig. 5a). Upon examination of responses to the higher doses over time, the encapsulated structure weight increased up to day 7, and then decreased slowly with time at both 100 mg and 300 mg doses (Fig. 5b). Experiments conducted with vehicle alone or with water soluble sodium pamoate at equivalent concentrations resulted in no FBR after 24 and 72 h (data not shown).

**Fig. 6:**
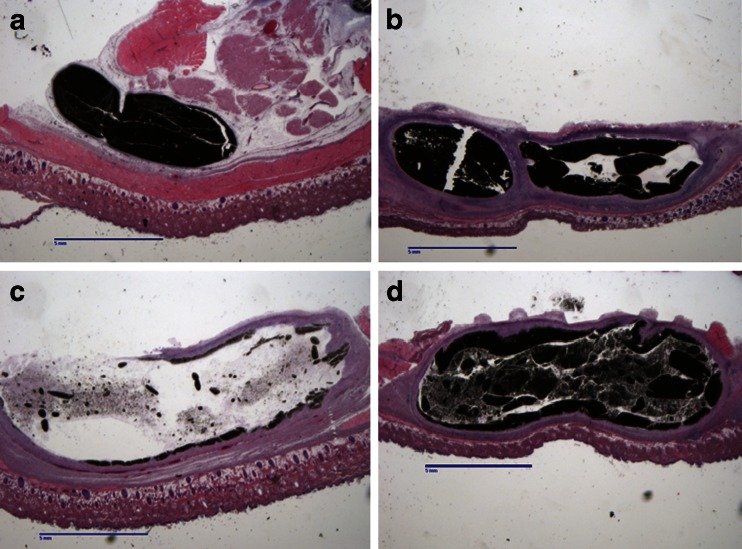
Histological observations of olanzapine pamoate monohydrate injection sites. Micrographs of the encapsulated structure and surrounding tissue, comprising the injection site, The IS excised from rats at (**a**) day 1, (**b**) day 7, (**c**) day 14, and (**d**) day 21 following SC administration of 300 mg of OLZ. The images are shown at 0.5× magnification following H&E staining. *Slides* are representative of four animals per time point.

The early response profile for OLZ is similar to that seen with APZ, with tissue and fluid surrounding the encapsulated structure on day 1 (Fig. 6a). However, while fibrous capsule is formed by day 3 (data not shown), negligible fluid or cellular debris is found within the structure associated with OLZ injection. The structures increase in size up to day 7 (Fig. 6b), at which point fluid and cellular debris are seen inside the structure. The wall of the structure, while fibrous and well vascularized, is thin and easily ruptured. The content includes material having the bright yellow color of OLZ (distinct from APZ, which is white in appearance). The structure begins to decrease in weight and fluid content after day 7, and formed into nodules by day 14 (Fig. 6c). This nodule is still present on day 21, but continues to decrease in size and throughout contains limited fluid (Fig. 6d). These observations demonstrate that while a similar tissue response is observed with OLZ (Fig. 5) and APZ (Fig. 1), there are key differences in terms of size, histological features, and rate of resolution.

### Key Inflammatory Cytokines Present in the FBR to Olanzapine Pamoate Monohydrate and in Surrounding Tissues

**Fig. 7:**
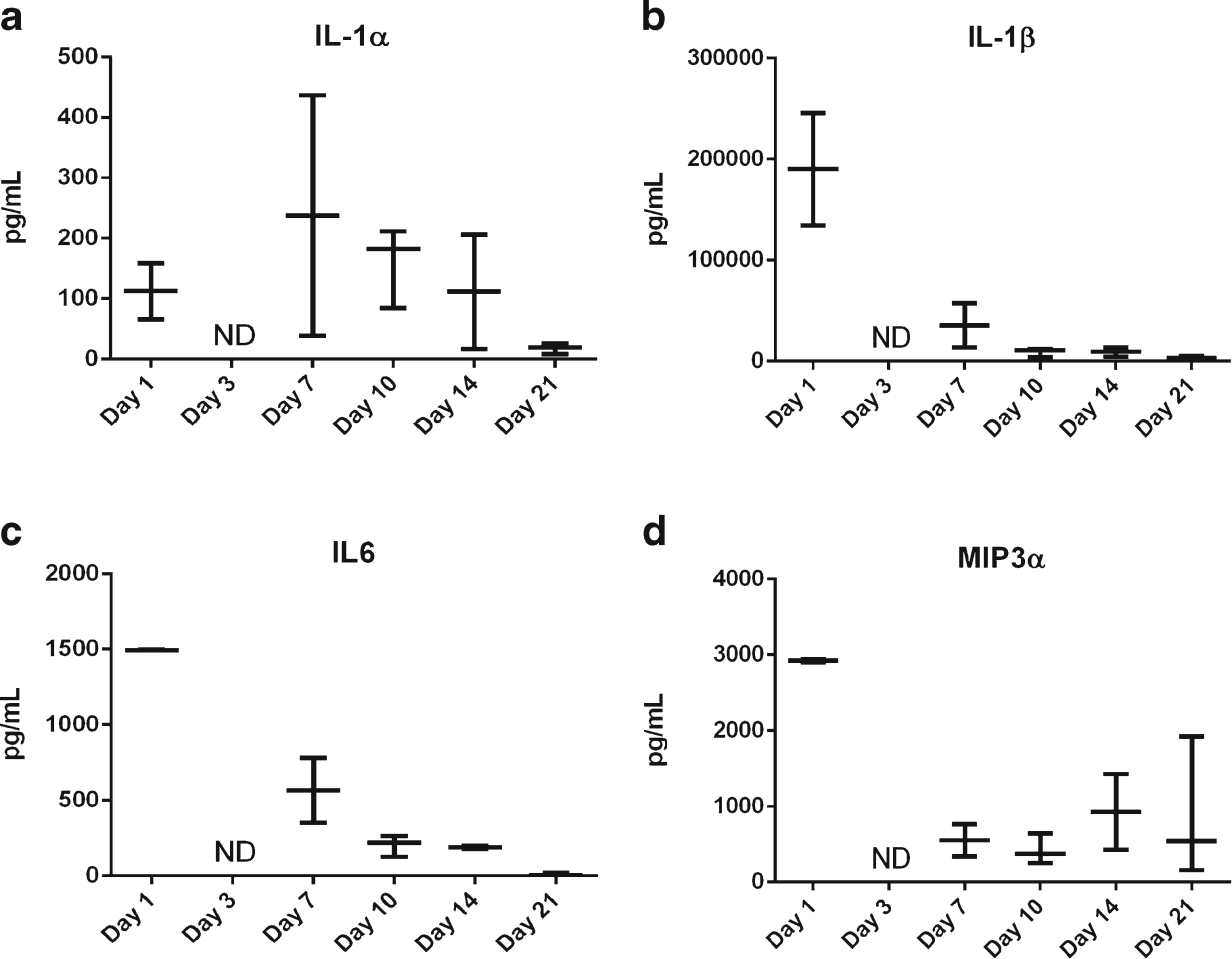
Cytokines involved in the ISR to olanzapine pamoate monohydrate. (**a**) IL-1α, (**b**) IL-1β, (**c**) IL-6 and (**d**) MIP3α cytokines present in encapsulated structure and surrounding tissue after subcutaneous injections of 300 mg olanzapine pamoate monohydrate. The injection site from day 3 contained no discernible fluid; therefore cytokine levels at the site could not be determined. Cytokine levels from each animal are plotted with bars indicating mean (*n* = 3 per time point and dose).

The differences seen in terms of the gross morphology of the response to APZ and OLZ may be due to differences in FBR cytokine expression. Therefore, multiplex analysis was performed on samples of the tissue surrounding the encapsulated structure resulting from injection of OLZ. While IL-1α levels are low (Fig. 7a) in the days following subcutaneous injection, the tissue around the encapsulated structure has high levels of IL-1β (Fig. 7b), IL-6 (Fig. 7c) and MIP3α (Fig. 7d). These elevated levels of IL-1β, IL-6 and MIP3α however are reduced overall from what is observed with APZ in the encapsulated structure. These early differences indicate that OLZ induces a FBR different from that induced by APZ. The differences presumably influence the morphology and duration of the tissue response to the foreign material.

**Fig. 8:**
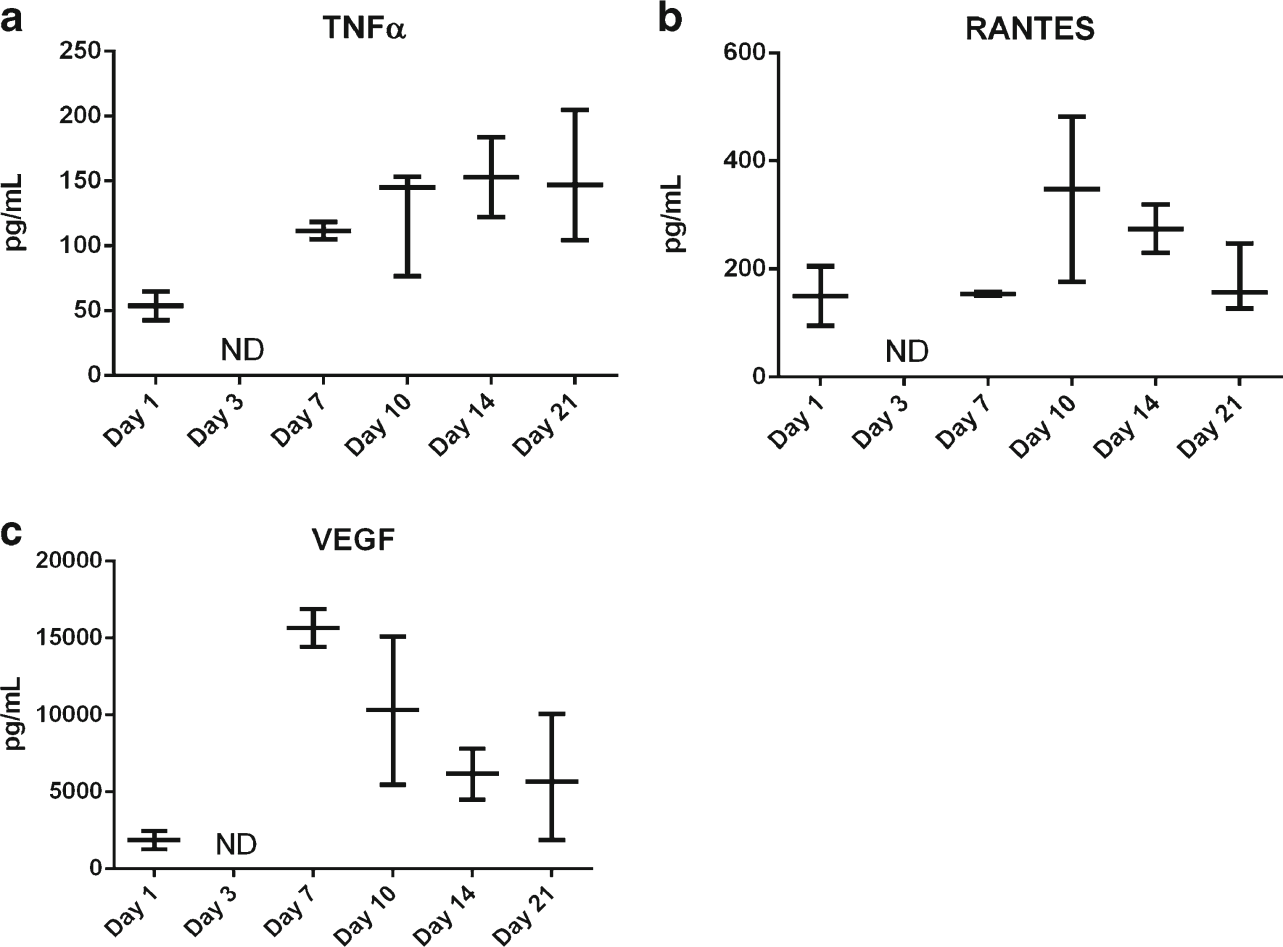
Later cytokines involved in the ISR to olanzapine pamoate monohydrate. Significant levels of (**a**) TNF and (**b**) RANTES are found at encapsulated structures and in surrounding tissue after 300 mg injection with 300 mg of the olanzapine pamoate salt. (**c**) VEGF is also elevated in the tissue surrounding the structure by day 7, but then declines over time. Cytokine levels from each animal are plotted with bars indicating mean (*n* = 3 per time point and dose).

The cytokines seen later in the response to OLZ are similar in type, but reduced in quantity compared to APZ response. Both TNFα (Fig. 8a) and RANTES (Fig. 8b) are elevated between days 7 and 10. TNFα levels continue to rise through day 21, although the overall response appears limited. RANTES levels peak at day 10, and decrease throughout the remainder of the observation period. VEGF was also present at the structure (Fig. 8c), although it is induced later in the response and exhibits peak levels at day 7. VEGF levels then steadily decrease over time in parallel with the decreasing size of the IS.

The pattern emerging from this data is that while the FBR formed as a result of subcutaneous injection with two poorly soluble ATAPs are similar in many respects, there are also key differences indicating that the tissue responds in a distinct manner to each compound. Given that material amounts were matched between the experiments with the two insoluble drugs, particle size distributions were in a similar ranges, and both are highly water-insoluble and thus resident and detectable in the sites for the duration of the experiment, it is likely that the details of the pharmacological profile of the deposited compound play a role in the response.

### FBR is a Localized Tissue Response Following Subcutaneous Dosing of ATAPs

**Fig. 9:**
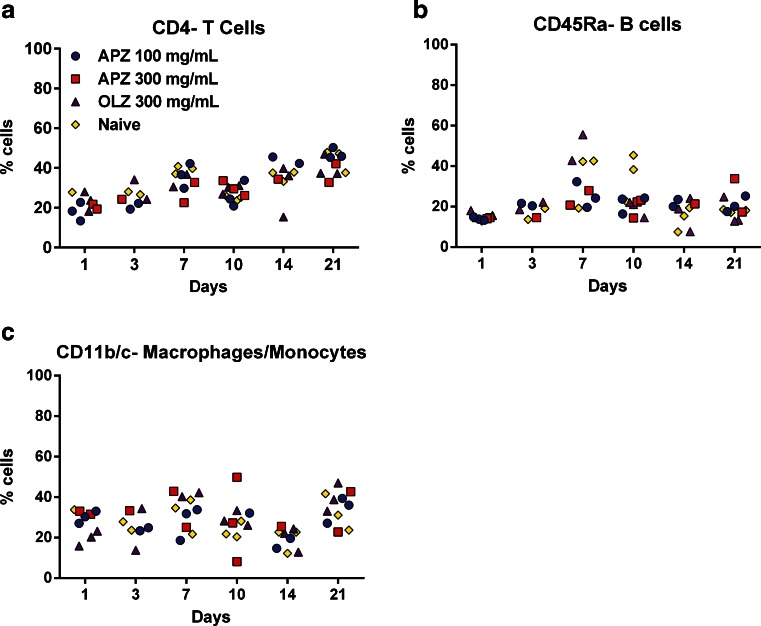
Systemic immune cell populations are not altered by aripiprazole monohydrate or olanzapine pamoate monohydrate. PBMCs were freshly isolated from animals on the noted time point, purified by density centrifugation, and stained with marker specific fluorescent antibodies and analyzed by flow cytometry. Cells were stained with (**a**) anti-CD4, (**b**) anti- CD45RA and (**c**) anti- CD11b/c. Data from each animal per group is plotted. ANOVA analysis was performed to determine significance.

The proteins induced at the site of injection are potent immunomodulators. If expressed systemically or released beyond the site of injection, the cytokines would have significant effects on the body. To evaluate whether the response was causing an alteration in circulating immune cells, which would indicate a systemic response to the compound, peripheral blood mononuclear cells (PBMCs) were isolated from the whole blood of rats that were injected with 300 mg APZ or OLZ and compared with control samples from naïve (untreated) animals. These cells were then stained with antibodies specific for several important immune cell markers and then analyzed by flow cytometry. Antibodies for CD4 (Fig. 9a), which is representative of the T-helper cell population, CD45 (Fig. 9b) as a marker for the B cell population, and CD11B/C (Fig. 9c) for macrophages/monocytes were used to look at general cell populations in both treated and untreated animals. Relative percentage of total cell populations was determined and compared to those of naïve animals.

While there are day to day variations in cell populations, no significant changes between treated and naïve animals can be detected by analysis of variance (ANOVA) (Fig. 7). Other cell types were also examined, including dendritic cells, neutrophils, CD8+ T cells and NK cells. No observable differences could be discerned between treated and naïve animals for these cell types (data not shown). Purified PBMCs from treated or naïve animals were examined *in vitro* for responsiveness to lipopolysaccharide in order to determine if there was any functional differences in these cells. No significant differences in IL-6 or TNFα secretion by these cells were observed, and there were no measurable differences in circulating serum cytokine levels in the animals as well (data not shown). These findings suggest that the FBR observed following subcutaneous dosing of an LAI ATAP does not elicit an immune activation beyond the IS. Based on this evidence, the immune response generated by poorly soluble ATAP injections is localized and does not impact the overall immune status of the animal.

## DISCUSSION

The studies described here establish a baseline approach for describing the foreign body response to LAIs which has not previously been demonstrated. Aripiprazole monohydrate (APZ) and olanzapine pamoate monohydrate (OLZ) were selected as model LAIs for the study of injection site and foreign body reactions in non-human primates and rats. The choice of drugs was based on therapeutic relevance, physico-chemical properties (these water-insoluble compounds both exhibit intrinsic extended release upon subcutaneous dosing), and the observed local tissue responses they generate (31). While in clinical practice these materials are injected intramuscularly, subcutaneous dosing presented significant advantages for the present study. Subcutaneous administration is ideal as a model on account of the relevance of the route for LAIs of various drugs, the immunological sensitivity of the SC space, accessibility and palpability of the injection sites, and because it allows for the administration of clinically relevant doses in total volume, not on a mg/kg basis. As illustrated by the non human primate data, the reaction that is seen in the subcutaneous space also mimics what may be seen in the intramuscular space in larger animals. Cynomolgus monkeys are frequently used for toxicology studies as their responses and metabolism to compounds closely resembles human responses. The data from this study demonstrate that there is a significant local tissue response to aripiprazole monohydrate at clinically relevant doses. Gross and microscopic pathology shows that there was chronic active inflammation throughout the duration of the study, indicating that the compound is highly irritating at the site, and continues to be irritating as long as it is present in the tissue. While this model validates clinical data available for many LAI compounds currently in use, it is not feasible to use for more detailed studies. Therefore, rats were used to further describe the chronic-active inflammation process in SC injection site.

Initial rodent experiments elucidated a dose relationship for the FBR, as measured by weight of injection site and surrounding tissue with increasing dose. Additionally, there appears to be a threshold dose, ≥100 mg, above which the tissue response, including the size of the encapsulated structure, increase disproportionately with increasing dose. This response is characteristic of a foreign body response, which is a distinct sterile immunological response to foreign materials or particles implanted in the body (6, 32). After the initiation of the FBR by the implantation of the material, soluble mediators such as chemokines and cytokines produced by infiltrating inflammatory cells and the surrounding tissue regulate the progression of the reaction (32).

Significant tissue responses are observed following SC administration of APZ and OLZ, which, based on this characterization study, represent a type of FBR. There are two specific phases to this FBR distinguished by an early, acute inflammation phase and a secondary, chronic inflammation phase. The acute phase is most likely triggered by the tissue damage resulting from the mechanical perturbation of the subcutaneous space and the presence of the drug particles in the depot. The cytokines present early in the response (IL6, IL-1α, IL-1β, MIP3α) indicate a potent immune response initially, which is characteristic of the acute-phase response triggered by tissue damage resulting primarily from the presence of foreign material. These cytokines may also help direct the later response to the depot and contribute to the formation of the fibrous capsule. The secondary phase of the response is characterized by local appearance of cytokines, chemokines, and proteins that work to control the response despite the continued presence of the foreign material. This chronic inflammation contributes both to the maintenance of the tissue response and the wound healing characteristics that ensue. The overall response demonstrated is similar to foreign body responses elicited by implanted materials such as naltrexone microspheres (33), collagen disks (34), and other implanted biomaterials (35).

The foreign body response (FBR) is part of the host response to tissue injury (1, 6). In this case, the FBR is being induced due to the subcutaneous injection of the ATAPs and the tissue injury resulting from this injection. The acute inflammatory response is characterized by fluid, proteins, and blood cells leaving the vascular system and infiltrating the site of injury. A fibrin matrix also forms at the site shortly after mechanical injury, influencing the second phase of the FBR, which includes the continued presence of inflammatory proteins, mononuclear cells and granulation tissue at the site, and can develop into a fibrous capsule (6).

TNFα, IL-1, and IL-6, key cytokines induced after injury, are elevated following administration of the ATAPs investigated. Mononuclear cells, characteristic of a chronic inflammation response, remain at the site of injection up to day 21, despite the formation of fibrous capsules, which are established during the wound healing process. This is the result of the persistent inflammatory presence of the ATAP at the site of injection. As long as the foreign material is present, the FBR will continue. However, once the compound has left the depot, the wound healing response can achieve resolution. For example, the FBR to the 300 mg aripiprazole suspension results in continued increase in mass at the injection site, while the lower dose has begun to resolve by day 21. At the higher dose, more of the compound is present, and due to the poor solubility and slow delivery rate from the site, the residence time of the material increases with dose.

It is known that shapes and sizes of a given material can affect the capacity of macrophages to attach to and engulf a foreign body (36). While crystal shapes and surface chemistries are inherent properties particular to a chemical composition, we have sought to size match the two ATAPs in our study so that their size distributions achieve two main criteria: (1) relevance to the product injected in each case (*e.g.* micron-sized crystals, not nano-crystals), and (2) particle sizes exceed that which macrophages can engulf. Prior experience with aripiprazole indicates that precipitates that form in injection sites are irritating, regardless of particle size control(31). We therefore conclude that the impact of mass injected and the chemical nature of the compound have a leading impact on the resulting FBR.

In comparison with injection sites containing APZ, the FBR to OLZ resolves at a faster rate. The kinetics and cytokines present at the site differ in ways that are not solely due to dose for mass adjustment, *i.e.* 300 mg OLZ, which is equivalent to 143 mg olanzapine-free base (correcting for molecular weight differences between the salt and the free base drug). If the tissue response were purely mass based, the FBR following SC administration of OLZ should roughly mimic that of APZ at 100 mg. Clearly this is not the case, as the response to 100 mg of APZ is aggressive in comparison to the 300 mg OLZ dose. Instead, other factors, such as solubility, lipophilicity, and resultant depot morphology following injection of the individual compounds may also contribute to the tissue response. The pharmacology of the active pharmaceutical ingredient (API) likely also influences the response. It has been observed that the chemical nature of the active drug component can be the cause of chronic inflammation although the mechanism is unclear (28, 33, 37). In these studies, it is evident that different APIs indeed lead to distinct FBR, but this aspect requires further investigation.

While there is a significant and potent FBR occurring at the site of injection involving key cytokines, a concomitant systemic immune response is not detectable. There are no detectable changes in the circulating immune cell population during the reaction and no elevation of circulating cytokines is seen (data not shown), further supporting the finding that the FBR is localized to the injection site and surrounding tissue. This is important to note, because unlike FBRs to inert inorganic materials, drug particles dissolve over time and the drug is absorbed into the body to elicit pharmacological effect remote from the injection site.

In summary, LAIs of ATAPs induce a significant but localized FBR at the site of injection and surrounding tissue. This response is characterized by an early acute response followed by a chronic inflammation phase. A surprising finding is that the response to the two ATAPs could be differentiated by both histopathology and by monitoring cytokine release at the site of injection, indicating that not all drugs in the same class are alike in this regard. Very little is known about the interplay between the physico-chemical properties of crystalline ATAPs and tissue dynamics at the site of injection. Future studies aimed at determining the mechanism of the response and the chemical/cellular interactions controlling the response may provide new insight in controlling the FBR and in designing the next generation of LAIs.

This work also establishes an experimental paradigm in determining events, magnitude, and timing at the site of injection from an immunological perspective. Currently, the description of these reactions in the literature is limited. Differences in the foreign body responses have been studied in other areas such as implants. This work is the first study to address and define the localized tissue reaction elicited by poorly soluble crystalline LAIs of atypical antipsychotics designed to reside in the tissue bed and release drug into the body for an extended period of time.

## ABBREVIATIONS

API: Active pharmaceutical ingredient
APZ: Aripiprazole monohydrate
ATAP: Atypical antipsychotic
CMC: Carboxymethylcellulose
FBR: Foreign body response
IS: Injection site
ISR: Injection site reaction
LAI: Long-acting injectable
OLZ: Olanzapine pamoate monohydrate
PBMC: Peripheral blood mononuclear cells
